# Pluripotent stem cells proliferation is associated with placentation in dogs

**DOI:** 10.1590/1984-3143-AR2020-0040

**Published:** 2020-08-06

**Authors:** Juliana Barbosa Casals, Naira Caroline Godoy Pieri, Kelly Cristine Santos Roballo, Fabiana Fernandes Bressan, Phelipe Oliveira Favaron, Daniele dos Santos Martins, Carlos Eduardo Ambrósio

**Affiliations:** 1 Departamento de Cirurgia, Faculdade de Medicina Veterinária e Zootecnia, Universidade de São Paulo, São Paulo, SP, Brasil; 2 Departamento de Medicina Veterinária, Faculdade de Zootecnia e Engenharia de Alimentos, Universidade de São Paulo, Pirassununga, SP, Brasil; 3 School of Pharmacy, University of Wyoming, Laramie, WY, USA

**Keywords:** stem cells, placenta, gestation, carnivores

## Abstract

Pluripotent stem cells have been studied as source of cells for regenerative medicine and acquire or genetic diseases, as an innovative therapy. Most tissues have stem cells populations, however in few quantities or impossible to be used during adult life, which lead to scientists look for new sources. Thus, this study aimed to analyze the presence of pluripotent cells in the uterus and placenta, following up non-pregnant, pregnant (begin, middle, and final), and postpartum periods in dogs. The uteri were obtained from social castration programs for population control in Pirassununga, Sao Paulo, Brazil. It was collected 20 uteri at different stages. The samples were fixed and processed for immunohistochemical analysis of NANOG, OCT4 and SOX2 expression, knowing as pluripotent stem cells makers. Our results showed positive expression for NANOG, OCT4 and SOX2 in all stages of gestation and nonpregnant uterus; however, we highlight some quantitative different between stages. OCT4 showed more expression in non-pregnant uterus than NANOG and SOX2, and its expression increased in pregnant uterus. In pregnant uterus there was more expression of NANOG than OCT4 and SOX2. Interesting, no difference was found between these markers in the other periods. In conclusion, it was possible to identify pluripotent stem cells in all periods in dog placenta and uterus, however during the early stage of pregnancy we observed more pluripotent stem cells than in all the others periods confirming the high plasticity and regeneration capacity of the uterine tissue.

## Introduction

The uterus is the main organ of the mammal’s reproduction, formed from Müller’s ducts in early embryogenesis (Dyce et al., 2010; Hyttel et al., 2010; Reece et al., 2013). During the gestational period, it is in close relationship with the embryo protecting and helping in the embryogenesis (Dyce et al., 2010; Hyttel et al., 2010; Reece et al., 2013). In carnivores, such as dog, the uterus is classified according to its form as bicornuate (Miglino et al., 2006; Furukawa et al., 2014).

Since the implantation to the birth of the conceptus, uterus is in interaction with fetus by sending and receiving information through the maternal fetal cell to cell communication (Miglino et al., 2006; Furukawa et al., 2014; Antoniadou and David, 2016). Thus, many researches are working on improve the knowledge of the mechanisms and cells involved on this organ remodeling during gestation, embryo development, and placenta formation (Miglino et al., 2006; Furukawa et al., 2014; Antoniadou and David, 2016).

The placenta is originated from the trophoblast layer, formed during early embryogenesis, and is composed on the most animals by yolk sac, amnion, allantois, and chorion. In carnivores the placenta is classified as choriovitelline during early embryo development and then chorioallantoic from middle to late development, also is identified as a zonary placenta (Miglino et al., 2006). The interaction between placenta and uterus is classified as endotheliochorial (Miglino et al., 2006; Furukawa et al., 2014).

The zonary placenta is composed by labyrinth, junctional and glandular zone (Furukawa et al., 2014). The labyrinth zone is composed by trophoblastic lamellae, with cytotrophoblasts and syncytiotrophoblasts covered by maternal vessels (Miglino et al., 2006; Furukawa et al., 2014).

Histological analysis of the endotheliochorial placenta shows maternal uterine epithelium interlaced with embryo epithelium and trophoblast migration to this region in direct contact with the maternal epithelium (Enders and Carter 2012). Endometrium and trophoblast play a critical role involving the maternal and embryonic cellular adhesion during the implantation process and further placentation (Miglino et al., 2006; Furukawa et al., 2014).

Stem cells have been the subject of several studies and have been growing the search for new sources of them (Verfaillie et al., 2002; Wang et al., 2005; Kuijk et al., 2011). Although, most tissues have stem cells populations, however they are in few quantities and sometimes in inaccessible places to be used in therapies (Zucconi et al., 2010; Wenceslau et al., 2011; Pelosi et al., 2011; Vidane et al., 2013; Roballo et al., 2013; Vidane et al., 2014; Gonçalves et al., 2014; Brólio et al., 2012; Borghesi et al., 2019). Which lead to scientists look for more viable sources of stem cells, for example in the placenta and uterus (Zucconi et al., 2010; Wenceslau et al., 2011; Pelosi et al., 2011; Vidane et al., 2013; Roballo et al., 2013; Vidane et al., 2014; Gonçalves et al., 2014; Brólio et al., 2012; Borghesi et al., 2019).

Nowadays, some researches have been involving mesenchymal progenitor cells from canine fetal tissues as yolk sac, umbilical cord vein, progenitor blood cells, and from cats as umbilical cord (Zucconi et al., 2010; Pelosi et al., 2011; Vidane et al., 2013, 2014; Brólio et al., 2012). For example, a recent study showed that it is possible to generate induced pluripotent stem cell form domestic animals, and proved that these cells have advantages, skills characteristics, and potential for applications in future cell based therapies (Gonçalves et al., 2012).

Furthermore, previous investigations have demonstrated that multipotent cells are present in amniotic membranes on human placenta (Yen et al., 2005). Although, the placenta harbors multiple types of different stem cells that still not well characterized, which implicates on its use in regenerative medicine (Levi and Morrison, 2008).

In humans, studies have described the relation of specific stem cells on the regeneration and maintenance of the endometrium during the follicular phase and embryo development (Matthai et al., 2006; Xu et al., 2011; Figueira et al., 2011; Łupicka et al., 2015). Furthermore, a study showed the detection of OCT4 positive endometrial cells, proving theirs pluripotential and presence of stem cells on this tissue, however these authors did not determinate the specific type of cells which expressed this pluripotent marker (Figueira et al., 2011).

During the development of placenta and embryo, factors as OCT4 and NANOG are expressed in both and they are characterized as important factors of pluripotency (Antoniadou and David, 2016). OCT4 was also already found in the inner cell mass against differentiation into trophectoderm (the embryonic contribution to the placenta) and it can be related to evolution of the mammalian placenta (Niwa et al., 2008).

Human embryonic stem cells (hESCs) pluripotency is regulated by several key transcription factors including NANOG, OCT4, and SOX2. Although the functions of OCT4 and NANOG in hESCs are well defined, however SOX2 has not been fully characterized (Adachi et al., 2010).

*SOX2* encodes a non-histone protein, capable of binding to DNA, influencing positively or negatively the expression of certain genes, and participates in the maintenance of self-renewal of undifferentiated embryonic stem cells. OCT4 participates in two crucial steps in the formation of the embryo, the morula stage and begin of the inner cell mass differentiation, and on the trophoectoderm development. NANOG is perhaps the most important one, because it acts mainly maintaining the self-renewal capacity of stem cells and their undifferentiated state (Puscheck et al., 2015).

In domestic animals there are few studies confirming the presence of stem cells populations on uteri and placenta. Thus, in our study we aimed to find and understand the behavior of these stem cells during the development of the placenta and the uterus before the after pregnancy by the expression of the most common pluripotent factors used, NANOG, OCT4 and SOX2, in different uterus status and placenta in dogs to knowledge the role of these proteins on the maintenance and plasticity of fetal membranes of gestation and uterus regeneration.

## Material and methods

### Tissue samples isolation

All procedures and protocols were entirely carried out according to the Committee of Ethics of the Veterinary Medicine School, University of Sao Paulo, Brazil (CEUA/FMVZ-Protocol number: 8402270515). The placenta was obtaining from healthy domestic dogs (n=20) with body weights classified as small (≤9 kg) or medium (>9 to 20 kg) from a program for dog population control in Pirassununga, Sao Paulo, Brazil. The uteri from the pregnant animals between 10 to 45 gestational days were collected, and divide in three groups classified by the gestation or no gestation stage: “nonpregnant” (n=4); “begin” (beginning) included uteri between 8 to 20 days of gestation (n=4), “middle” between 21 to 30 days of gestation (n=4) and “final” between 31 to 60 days of gestation (n=4) and “postpartum” (n=4).

The gestational aged was estimated by organogenesis analysis and measurement using crown-rump length (CRL) of the embryos (Evans and Sack, 1973; Pieri et al., 2015). The uteri were dissected and fixed in paraformaldehyde 4% (pH 7.4–0.1 M), dehydrated and embedded in paraffin, and then, serial sections were made. The slides were analyzed using microscope Zeiss KS400 (Oberkochen, Germany) and images were captured by AxiVision 4.7.7 software.

### Immunohistochemistry

**Figure 1:**
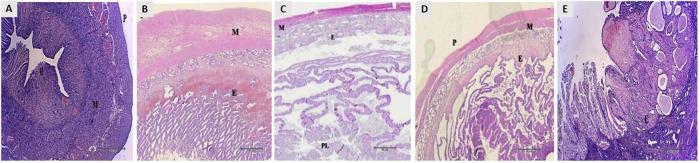
Morphological changes before to after pregnancy. Nonpregnant uterus (A), begin (B), middle (C) and final (D) of pregnant uterus, and postpartum (E). Endometrium (E), myometrium (M), placenta (PL) and perimetrium (P) in canine uterus. Bar: 20μm.

Immunohistochemistry analysis was conducted on sectioned uterus and placenta to determine the spatial distribution of the stem cell marker-positive cells following the protocol from Roballo et al. (2013) with some modifications. The following antibodies were used: OCT4, SOX2 and NANOG polyclonal anti-rabbit (1:200, Ab18976, Ab97959, Ab80892, Abcam, Cambridge, England, United Kingdom). The sliced samples were boiled in citrate buffer (0.01M; pH 6) in microware for 20min to recover antigens. The material was then cooled in room temperature and treated with HCl in 38.5°C for 20 min. The slides were incubated with 0.6% hydrogen peroxide (H_2_0_2_) in methanol for 45 min, for blocking endogenous peroxidases. Non-specific binding block was perfumed by Dako for 10 min. Primary antibody was omitted as a negative control (Figure 1). The slides were washed three times TBS containing 0.5% Triton X-100 (TBST), and incubated Kit Dako advance (DAKO ADVANCE™ HRP). The immunohistochemistry reaction possible to be identified by DAB (diaminobenzidine-Sigma) peroxidase substrate Kit (3.3 diaminobenzidine tetrahydrochloride) and counter stained with hematoxylin. Previously tested mice brain, and canine embryos were used as positive controls for the antibodies.

### Cells counting

For measure the expression of NANOG, OCT4, and SOX2 antibodies in the cells from all uterus stages, histological images were observed at 40x magnification objective, in an optical microscope (Leica DM 2500, Berlin, Germany), and captured using Axion Vision software (Zeiss). From each sample, 10 random fields of 10 captured images were used, and a total amount of positive cells from these fields was counted.

### Statistical Analysis

Percentage of positive cells of each marker on each period was calculated by ANOVA – Tukey´s multiple comparisons test. The statistic test was realized by software GraphPad Prism4® (p< 0.05).

## Results

The 20 canine uteri were analyzed by histology and immunohistochemistry for the presence of pluripotent markers (NANOG, OCT4, and SOX2), through five timelines, and positive cells for each antibody on each period was quantified, followed by statistical analyzes.

Histological analysis showed differences on the epithelia on all periods, the nonpregnant uterus was closed with the myometrium preserved (Figure 1A), during the pregnancy the uterus presented expressive modifications on its epithelium (Figure 1BD), and postpartum uterus presented a recovered aspect (Figure 1E).

**Figure 2:**
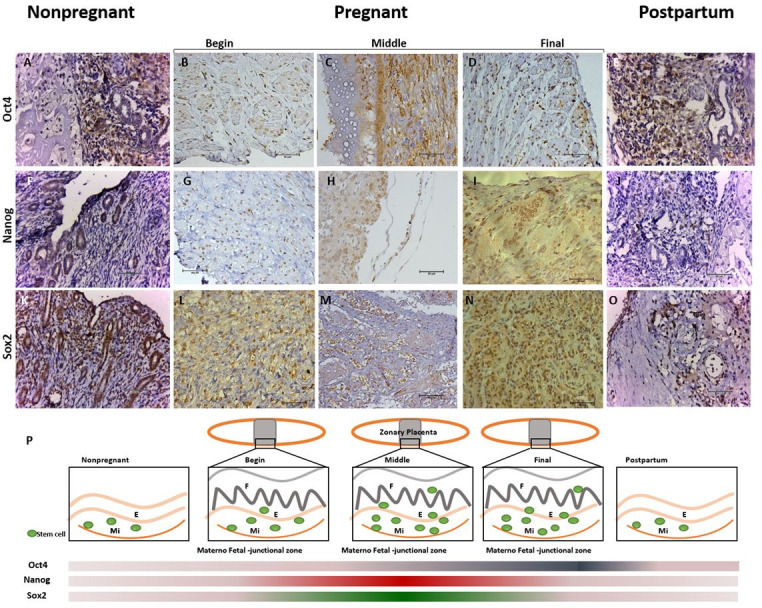
Expression of NANOG, OCT4 and SOX2 in canine nonpregnant, pregnant and postpartum uterus. Expression of pluripotent markers in the endometrium (**A-O**). SOX2 was expressed in the epithelium of the endometrium and in the glandular epithelium (**K-O**), OCT4 was expressed in the myometrium (**A-E**) in nonpregnant uterus. NANOG and SOX2 were expressed in the endometrium on the beginning of pregnancy (**A, F, K**). OCT4 and NANOG were expressed in the myometrium and SOX2 in the endometrium on the middle of pregnancy (**C, H, M**). OCT4 was expressed in myometrium, NANOG in endometrium and SOX2 in the laminar region on the final period of pregnancy (**D, I, N**). NANOG, OCT4 and SOX2 were spread on the begin of the pregnancy (**B, G, L**). All markers were expressed in myometrium and endometrium on the postpartum period. **(P)** Summarizing, OCT4 and NANOG were expressed more on middle of pregnancy and SOX2 on final period. However NANOG and SOX2 had a pick of expression on the middle of the pregnancy, and OCT4 was more expressed from the middle to final of pregnancy. (Mi= myometrium; E= endometrium; F= fetal tissue) (Bar: 20μm).

The immunohistochemistry demonstrated positive cells staining during all groups for all pluripotent markers (Figure 2AO), on all regions of the uterus walls, without a specific region of concentration, and spread on all layers. In the pregnant uteri, the belt region was the selected area of analysis, since it is the most interaction region between maternal and fetal epithelia, and it was observed also positive cells on all regions included fetal tissues.

It was possible to observe OCT4 staining in all stages, however, with more positive cell during the gestational period (Figure 22P) in average (104.9±15.66) and postpartum (91.2 ±12.07). It was possible to observe NANOG and SOX2 staining in all stages, however, with more positive cell during the gestational period (Figure 22P) in average 128.4 ±10.19 and 116.8 ±9.54 (Table 1). The expression of NANOG and SOX2 was evident in a large proportion of the cells from perimetrium, myometrium and endometrium, and in some stages of pregnancy it was possible to find as well on the villi of the fetal chorion.

**Table 1:** Means and standard deviation of average of the number of positive cells for the pluripotent markers OCT4, NANOG and SOX2 between gestational or non-gestational periods in canine uterus.

**Pluripotent Markers**	**Nonpregnant**	**Gestation period**	**Postpartum**
**OCT4**	78.0 (±10.56)	104.9 (±15.66)	91.2 (±12.07)
**NANOG**	49.8 (±11.45)	128.4 (±10.19)	84.2 (±11.30)
**SOX2**	60.2 (±5.54)	116.8 (±9.54)	90,0 (±16.63)

The nonpregnant and postpartum uteri showed positive cells for all pluripotent markers, also spread on all layers without a determinate region. However, when we compared the expression between each marker, OCT4 was more expressed than NANOG and SOX2 (Figure 33E, and Table 1).

**Figure 3:**
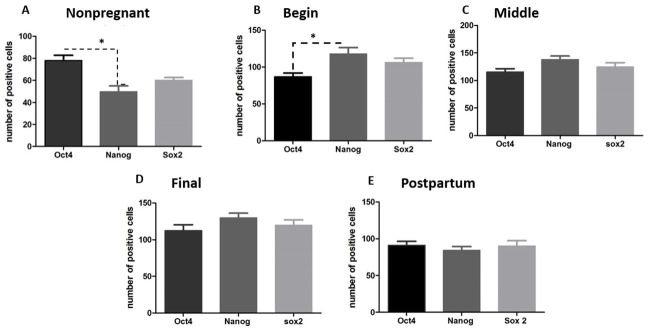
Graphs showing the expression of NANOG, OCT4 and SOX2 in the uterus of nonpregnant female dogs, pregnant (begin, middle and final of gestation), postpartum. (**A, B**) There are statistical difference between the number of positive cells for OCT4 and NANOG at nonpregnant and begin periods (p>0.05); (**C, D e E**) There are not statistical difference between the markers at middle, final and postpartum periods. ("*" means p< 0.05).

The junctional zone (belt in the zonary placenta) was analyzed during the begin, middle, and final period of the gestation. It was clear that the proportions of positive cells per marker were different, and the positive cells were concentrated on the labyrinth zone, where the maternal and fetal epithelium are in strong interaction. The labyrinth zone is composed by trophoblast cells, organized in lamellae, in which cytotrophoblasts and syncytiotrophoblasts are in interaction with the maternal supply, as the principal maternal fetal region for supply exchange.

**Figure 4:**
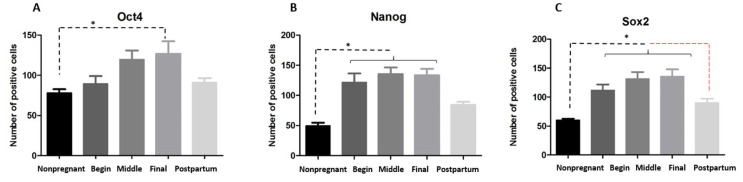
Analysis of the pluripotent markers’ expression on uterus during the periods proposed. (**A**) OCT4 was statistical difference between nonpregnant and final of gestation; (**B**) NANOG had significant difference between nonpregnant and pregnant uterus (begin, middle and final of gestation); (**C**) SOX2 had significant difference between nonpregnant and pregnant periods (begin, middle and final of gestation); postpartum and pregnancy (final of gestation) ("*" means p< 0.05).

After quantification of the number of positive cells in the gestational periods, there was difference in level of positive cells between NANOG and OCT4 (Figure 3B). The difference on the expression of NANOG and SOX2 was not statistically different (Figure 3AE). However, OCT4 (Figure 4A), NANOG (Figure 4B) and SOX2 (Figure 4C) detection was higher on pregnancy than nonpregnant and postpartum stages (Figure 2P), and NANOG and SOX2 were more expressed during this period than OCT4. OCT4 expression increased to the middle to final period and kept its expression on the postpartum (Figure 2P).

## Discussion

The canine placenta is histologically composed by the labyrinth, junctional and glandular zone (Furukawa et al., 2014). The placenta is in contact with the maternal epithelium since begin of the gestation, resulting in a straight exchange between embryo and mother thought tissues and vessels. The zonary placenta has a belt which is in a close relationship between fetus and maternal tissue, what can be observed in detail in the histology analysis. In our study we observed all these anatomic structures, already studied by other authors (Miglino et al., 2006; Furukawa et al., 2014).

Studies about stem cells presence in the uterine epithelium have been performed in humans as a source of potential cell therapies (Ono et al., 2010; Gargett et al., 2012; Yamauchi et al., 2020). Furthermore, studies with animal models show that endometrial cells have different proliferative capacity especially on the endometrial epithelium, suggesting the presence of stem cell population in this region (Chan and Gargett, 2006). In rodent’s placenta the presence of epithelial progenitor cells, which were able to self-renewal and to differentiate were already identified. These cells were in small regions and quantity and were hormone responsive (Janzen et al., 2013). In our study, we identified cells expressing stem cell markers that can be related with self-renewal of the endometrium and are probably is a stem cell population present in the uterus, and in relatively small quantity in accordance with the previous studies.

Herein we identified and confirm the pluripotent stem cell population on the uterus and placenta using the most common pluripotent markers, NANOG, SOX2 and OCT4 in accordance with the studies cited above (Janzen et al., 2013; Portmann-Lanz et al., 2006). We verified whether during the nonpregnant, pregnant and pos parturition periods, the presence and maintenance of these stem cells could be affected and if there were any correlation between the quantity of stem cells and the stage of the uterus.

We observed difference regarding the antibody detection before and after pregnancy. In the nonpregnant uterus, there were some pluripotent cells, however, fewer than in the pregnant uterus. OCT4 was more expressed during the middle to the final of the pregnancy, and NANOG and SOX2 were highly expressed during the whole pregnancy. This result leads us to affirm that during the gestational period the uterus needs more cell/stem cell support for regeneration and maintenance, and the stem cells present on it may be part of the uterus remodeling and regeneration. It is important to point out that our results are in accordance with studies performed with humans (Janzen et al., 2013; Portmann-Lanz et al., 2006).

In human myometrial tissue was also studied, as in our study, and OCT4 positive cells were confirmed and some of the cells resemble progenitor cells (Ono et al., 2010; Portmann-Lanz et al., 2006). In human placenta recently it was shown the presence of adult stem cell niche (Izumi et al., 2009; Antoniadou and David, 2016). Even though human and dogs do not have the same type of placenta, these previous findings are in accordance with our results in canines.

In humans, as well, a decrease of OCT4, NANOG and SOX2 expression was observed in the amnion on the final period of pregnancy when compared with the amnion from the fetus (Izumi et al., 2009; Chang et al., 2010). Our detection of pluripotent proteins showed an increase during the begin of the pregnancy thought final period, follow by a decrease from the final to the pos partition uterus; however, we analyzed the uteri and all the placenta, however, the amnion was not analyzed as the study above.

OCT4 transcript factors are correlated with cell pluripotential and can be expressed on cells lineages as embryonic stem cells, induced pluripotent stem cells and most of studies with placenta analyzed OCT4 expression only in the amnion membrane (Lee et al., 2006). On our study we analyzed all the layers and tissues of the placenta, and we observed OCT4 expression in the canine zonary placenta in the belt region and maternal fetal junction.

Lastly, in our findings NANOG was more expressed on endometrium and myometrium, and OCT4 was more expressed on endometrium and the maternal fetal junction. Studies in bovine described OCT4, NANOG and SOX2 expression more in the endometrium (Xu et al., 2011) and studies in humans showed more OCT4 positive cells in the endometrium (Figueira et el., 2011), thus both studies corroborating with our results.

## Conclusion

In conclusion, we identified pluripotent stem cells in all gestation periods in the canine placenta, and in nonpregnant and postpartum uteri by the presence of positive cells for NANOG, OCT4 and SOX2 markers. During the gestational periods there was an increase of pluripotent cells quantity, which lead us to confirm the function of stem cells niches in the tissue as a regenerative cell source. The future use of these cells in clinical trials will depend of more researches involving generation of standard protocols for cell isolation, culture and expansion; definition *in vitro* and *in vivo* of the biology function of these cells; and standardization of safety protocols and dose response of these cells in regenerative therapies. However, it is important to highlight that when more undifferentiated is the cell, greater its potential to be use in cell therapies applied to regenerative medicine.
